# Comparison of the Accuracy of Papanicolaou Test Cytology, Visual Inspection With Acetic Acid, and Visual Inspection With Lugol Iodine in Screening for Cervical Neoplasia in Southeast Nigeria

**DOI:** 10.1200/JGO.17.00127

**Published:** 2018-02-28

**Authors:** John Egede, Leonard Ajah, Perpetus Ibekwe, Uzoma Agwu, Emmanuel Nwizu, Festus Iyare

**Affiliations:** **John Egede**, **Perpetus Ibekwe**, **Uzoma Agwu**, **Emmanuel Nwizu**, and **Festus Iyare**, Federal Teaching Hospital, Abakaliki, Ebonyi State; and **Leonard Ajah**, University of Nigeria, Ituku-Ozalla Campus, Enugu, Enugu State, Nigeria.

## Abstract

**Purpose:**

This study aimed to compare the accuracy of Papanicolaou test cytology, visual inspection with 5% acetic acid (VIA), and visual inspection with Lugol iodine (VILI) in the detection of premalignant and malignant lesions of the cervix.

**Materials and Methods:**

This was a cross-sectional comparative study of 200 consenting participants at the Federal Teaching Hospital, Abakaliki over a 6-month period. All the participants had Papanicolaou test cytology. Subsequently, they were classified into two groups of 100 each through systematic random sampling: group 1 had VIA and group 2 had VILI. Thereafter, all the participants had cervical punch biopsy at the 6 and 12 o’clock cervical positions. Cervical punch biopsy was also done on the suspicious lesions of the cervix irrespective of their positions. The tests of validity of the three methods were calculated using the histology of the biopsy specimen as the gold standard. *P* value ≤ .05 was considered to be statistically significant.

**Results:**

Among the VIA group, 19 (19%) had cervical epithelial abnormalities on Papanicolaou test cytology, and VIA was positive in 14 (14%). Histology results showed cervical neoplasia in 15 (15%) of the participants. Among the VILI group, 15 (15%) had cervical epithelial abnormalities on Papanicolaou test cytology, and VILI was positive in 19 (19%). Histology results showed cervical neoplasia in 15 (15%) of the participants. There was no significant difference in overall accuracy of Papanicolaou test cytology, VIA, and VILI. The overall accuracy of the Papanicolaou test cytology plus VIA was significantly more than Papanicolaou test cytology alone.

**Conclusion:**

VIA or VILI can be used as a stand-alone cervical cancer screening test when compared with Papanicolaou test cytology, particularly in resource-limited settings. VIA can also complement Papanicolaou test cytology.

## INTRODUCTION

Cervical cancer is a major public health threat to women in many low- and medium-resource countries, including those in sub-Saharan Africa. It is the leading cause of cancer-related death among women.^1,2^ Cervical cancer is the second most common cancer of women worldwide, after breast cancer, and it remains the most common genital tract cancer in the developing countries.^3,4^ In Nigeria, the crude incidence and age-standardized incidence of cervical cancer are 17.1 of 100,000 and 29.0 of 100,000 women, respectively.^5^ Current estimates indicate that every year, 14,089 women are diagnosed with cervical cancer, and 8,240 die as a result of the disease.^5^ A woman’s lifetime risk of developing and dying as a result of invasive cancer in Nigeria is 2.1% and 1.7%, respectively.^6^ The high cervical cancer burden in Nigeria is because access to screening services for cervical cancer is often limited or nonexistent—thus, the continued increase in the disease burden.^7^

Cervical cancer is a preventable disease, because it has a preinvasive phase that can be detected and treated if women are screened. Effective regular screening programs for early detection and treatment of precancer lesions can lead to a significant reduction in the morbidity and mortality associated with this cancer and has been shown to reduce the risk of developing cervical cancer by 80%, as observed in developed countries.^8,9^ The mainstay of this preventive measure is the use of cervical cytology screening by the Papanicolaou test, which is recommended starting at the age of 21 years and should be done from once a year to once every 5 years, in the absence of abnormal results.^7,8,10,11^ In developed countries, regular Papanicolaou test screening has resulted in 60% to 90% reduction in the mortality associated with the disease.^12,13^

However, only approximately 5% of eligible women in the developing countries undergo cytology-based screening.^14^ Cytology-based screening programs are difficult to organize in low-resource settings owing to paucity of infrastructure, trained manpower, and funds, because the scarce resources are often channeled to presumed more pressing reproductive health issues.^10^ More importantly, in virtually all developing countries, cytology-based screening services are confined to teaching hospitals or private laboratories in urban areas, and delays in reporting cytology results make it less likely that women who test positive ever receive their results, let alone treatment or follow-up. These are some of the barriers that prevent cytology-based screening programs from being effective in developing countries.^15^ Therefore, there is a need for viable, accurate, inexpensive, and effective alternative screening methods for the control of cervical cancer in countries with limited resources, such as Nigeria.^16,17^ In settings where health care resources are scarce, screening efforts should be directed toward cost-effective strategies that are affordable and reliable.^18^ Studies have demonstrated that visual inspection of the cervix with acetic acid (VIA) or visual inspection with Lugol iodine (VILI) are alternative screening methods.^18,19^ They are cheap and noninvasive and can be done in a low-level health facility.^20^ More importantly, VIA and VILI provide instant results, and those eligible for treatment of the precancerous lesions can be treated immediately. This see-and-treat method ensures adherence to treatment soon after diagnosis, hence stemming the problem of default to appointment and referrals.^21,22^ On the basis of a Medline search, there was a paucity of studies in the subject matter, and no previous study in Nigeria compared the overall accuracy of Papanicolaou test, VIA, and VILI. This study was aimed at comparing the overall accuracy of VIA, VILI, and Papanicolaou test cytology among the eligible women attending the gynecologic clinic at Federal Teaching Hospital, Abakaliki (FETHA).

## MATERIALS AND METHODS

### Study Area

Abakaliki is the capital of Ebonyi State. Ebonyi State is one of the five southeastern states of Nigeria. It is mainly inhabited by the Igbo-speaking community and has a population of approximately 2.18 million, according to the 2006 population census.^23^ FETHA is a tertiary institution serving the people of Ebonyi State and their neighbors. The hospital has a well-developed obstetrics and gynecology department, with an established cancer-screening center.

### Study Design

This was a cross-sectional comparative study involving apparently healthy women who attended the gynecologic clinic at FETHA between September 2015 and February 2016. The women were counseled on cancer of the cervix, the screening procedures, and objectives of the study, after which consent to participate in the study was sought and obtained. The women were classified into two groups of 100 each through systematic random sampling. Each of the eligible participants was asked to pick a number that was sealed in a brown envelope. Those who picked 1 were allocated to group 1 and those who picked 2 were allocated to group 2. Papanicolaou test cytology was performed on all the participants, and the Bethesda system was used to report the results. Subsequently, those in group 1 had VIA and those in group 2 had VILI.

Application of 5% acetic acid on the cervix causes swelling of the epithelial tissue and reversible coagulation or precipitation of the cellular proteins. When 5% acetic acid is applied to normal squamous epithelium, little coagulation occurs, and it appears as pink on VIA. Cervical neoplastic cells have high coagulation on application of 5% acetic acid because of their high nuclear protein content, and they appear as acetowhitening on VIA. For the purpose of this study, VIA is positive when there is acetowhitening of the cervical epithelium after application of 5% acetic acid, and it is negative when there is no acetowhitening. Normal cervical squamous cells have enough glycogen, and cervical neoplastic cells have little or no glycogen. Iodine solution is glycophilic, and thus its application results in uptake of iodine in glycogen-containing epithelium. Therefore, normal cervical squamous epithelium stains mahogany brown or black after application of iodine, whereas cervical neoplastic cells appear thick mustard-yellow or saffron colored on VILI. For the purpose of this study, VILI is positive when the cervical squamous cells appear mustard yellow or saffron colored after application of Lugol iodine, and it is negative when the cervical cells appear mahogany brown or black. Cervical punch biopsy was performed at the 6 and 12 o’clock positions with Tischler biopsy forceps for all the participants. In addition, cervical punch biopsy was performed at the lesions for the participants with suspicious lesions on VIA or VILI, irrespective of their site of location in the cervix. The histology of the biopsy specimens was used as the gold standard in this study. The histology results were reported as normal; inflammation; cervical intraepithelial neoplasia 1, 2, or 3; or cervical malignancy. The Papanicolaou test, VIA, VILI, and cervical punch biopsies were performed by three gynecologists, and the cytology and histology of the specimen were performed by two histopathologists. The exclusion criteria were age younger than 21 years, history of abnormal results from previous cervical cancer screening, any visible cervical mass/lesion before VIA or VILI, pregnancy, and within 6 weeks postpartum. Also excluded were women with history of bleeding par vaginam and those who had coitus or douching within 3 days before screening.

The minimum sample size for the study was calculated on the basis of the formula for estimating sample size for cross-sectional studies.^24^ With 11.2% prevalence of cervical squamous cell abnormality previously reported at the study center,^25^ and adding a 10% attrition rate, the minimum sample size for the study was 168. A semistructured questionnaire was used to collate information from each of the eligible participants on the sociodemographic characteristics, clinical findings at pelvic examination, results of VIA or VILI, Papanicolaou test cytology, VIA plus Papanicolaou test cytology, VILI plus Papanicolaou test cytology, VIA plus VILI, and histology of the biopsy specimen. Data were analyzed with Statistical Package for Social Sciences version 20 software (IBM SPSS, Chicago, IL). Categorical variables were analyzed using the Pearson’s χ^2^ test. The level of significance was set at ≤ .05. The ethical clearance for this study was obtained from the Ethics Committee of FETHA.

## RESULTS

A total of 200 eligible women participated in the study. All the participants were Ibos and Christians. The mean age of the participants was 41.6 years. Table 1 lists comparison of the sociodemographic characteristics between the two groups of participants. There was no statistically significant difference between the two groups of participants on the sociodemographic characteristics. Table 2 lists the results of the Papanicolaou test cytology, VIA, VILI, and histology diagnoses among the study participants. Among the VIA group, 19 (19%) had cervical epithelial abnormalities on Papanicolaou test cytology, and VIA was positive in 14 (14%). Histology results showed cervical neoplasia in 15 (15%) of the participants. Among the VILI group, 15 (15%) had cervical epithelial abnormalities on Papanicolaou test cytology, and VILI was positive in 19 (19%). Histology results showed cervical neoplasia in 15 (15%) of the participants. Table 3 lists the correlation of Papanicolaou test cytology, VIA, and VILI with histology results among the study participants. Among the VIA group, Papanicolaou test cytology was positive in 19 (19%), and VIA was positive in 14 (14%) of the participants. Histology result was positive in 15 (15%) of the participants. Among the VILI group, Papanicolaou test cytology was positive in 15 (15%), and VILI was positive in 19 (19%) of the participants. The histology result was also positive in 15 (15%) of the participants. Table 4 lists the influence of age on the histology results of the participants. It showed that cervical neoplasia was more common among the older women (*P* = .03). The tests of validity of the screening methods used in detection of cervical epithelial abnormalities are listed in Table 5. Among the VIA group, sensitivity, specificity, positive predictive value (PPV), negative predictive value (NPV), and overall accuracy of Papanicolaou test cytology, VIA, and Papanicolaou test cytology plus VIA ranged from 58.3% to 97%. Among the VILI group, sensitivity, specificity, PPV, NPV, and overall accuracy of Papanicolaou test cytology, VILI, and Papanicolaou test cytology plus VILI ranged from 57.9% to 100%. Table 6 lists the comparison of overall accuracy of the screening methods used in detection of cervical epithelial abnormalities. With the exception of Papanicolaou test cytology plus VIA, which was found to be significantly more accurate than Papanicolaou test cytology alone, there was no statistically significant difference on overall accuracy among the other procedures.

**Table 1:**
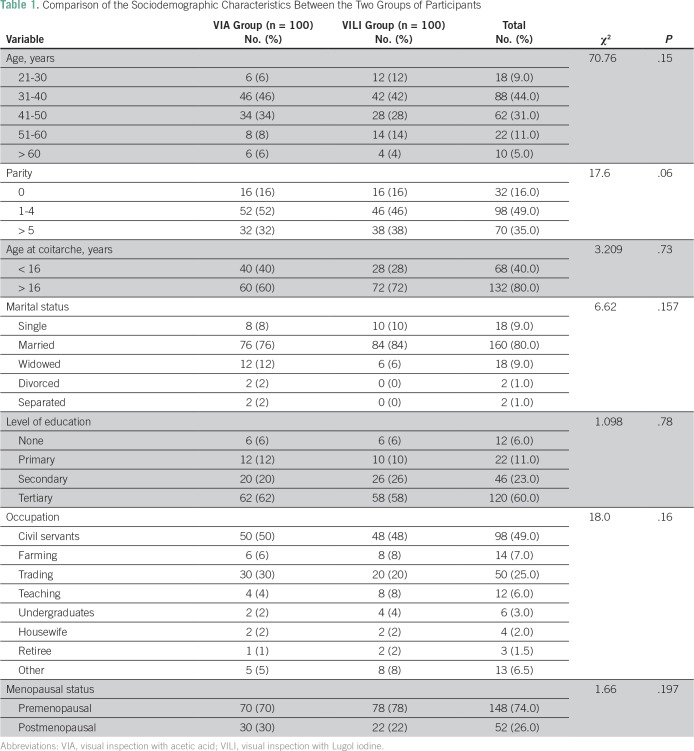
Comparison of the Sociodemographic Characteristics Between the Two Groups of Participants

**Table 2:**
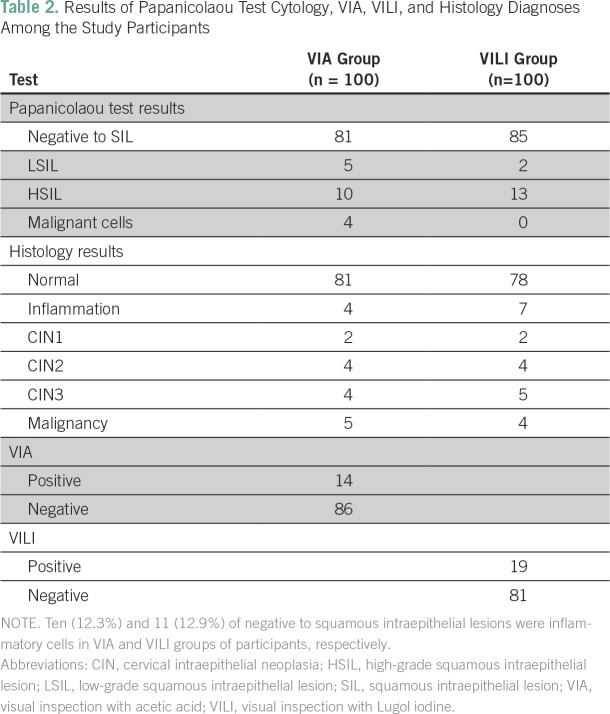
Results of Papanicolaou Test Cytology, VIA, VILI, and Histology Diagnoses Among the Study Participants

**Table 3:**
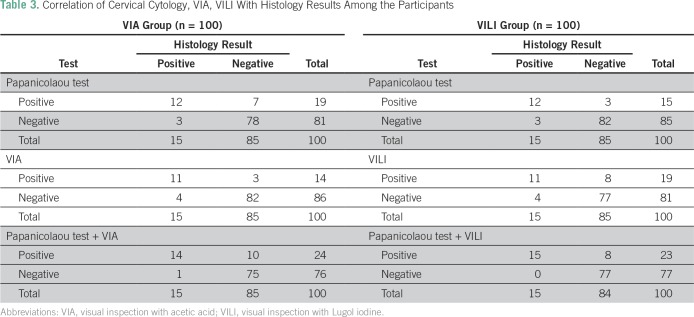
Correlation of Cervical Cytology, VIA, VILI With Histology Results Among the Participants

**Table 4:**
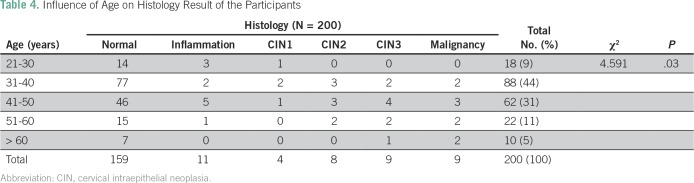
Influence of Age on Histology Result of the Participants

**Table 5:**
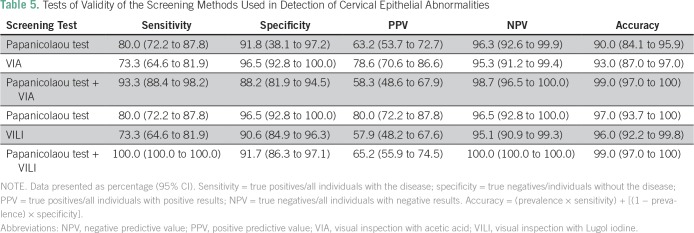
Tests of Validity of the Screening Methods Used in Detection of Cervical Epithelial Abnormalities

**Table 6:**
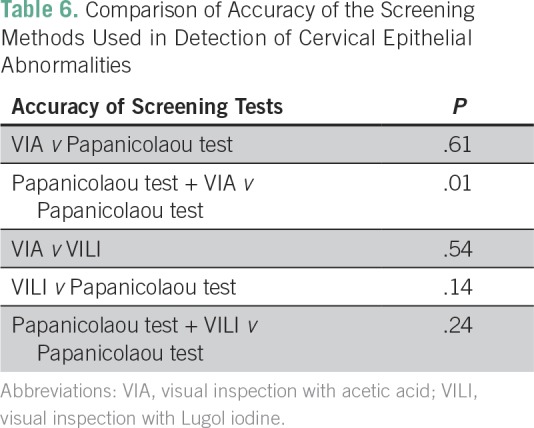
Comparison of Accuracy of the Screening Methods Used in Detection of Cervical Epithelial Abnormalities

## DISCUSSION

This study showed that there was no statistically significant difference in overall accuracy among Papanicolaou test cytology, VIA, and VILI. Although the combination of Papanicolaou test cytology and VIA was more accurate than Papanicolaou test cytology alone, there was no statistically significant difference between the combination of Papanicolaou test cytology and VILI and Papanicolaou test alone in overall accuracy.

The sensitivity and specificity of Papanicolaou test cytology reported in this study were essentially similar to the previous reports in India and in Kano, Nigeria.^18,26^ The 73.3% sensitivity of VIA reported in this study is similar to the previous reports in Kano, Nigeria and within the range recorded in a multicenter study in India and Africa.^26,27^ The tests of validity of VIA being essentially comparable to that of Papanicolaou test cytology in this study is similar to the previous reports by Omole-Ohonsi et al^26^ in Nigeria, Abdel-Hady et al^28^ in Egypt and Sankaranarayanan et al^29^ in India. The high NPVs of Papanicolaou test cytology, VIA, and VILI in this study suggest that whenever any of the test results is negative, the women can be reassured and sent home safely. This therefore suggests that in well-trained hands, VIA and VILI are affordable alternative methods of cervical cancer screening when compared with Papanicolaou test cytology. The combination of VIA and Papanicolaou test cytology, which improved the overall accuracy of the combination in this study, is similar to the previous reports.^2,30^ This suggests that both tests would detect more cases than Papanicolaou test cytology alone. Thus, in a low-resource setting like ours, VIA can be used in two ways: either by replacing the Papanicolaou test cytology for universal screening or by being complementary to Papanicolaou test cytology for opportunistic screening.

The sensitivity and specificity of VILI in this study are comparable to the findings of the multicenter studies done in India and Africa.^2,27^ The lower specificity of VILI compared with that of VIA in this study differed from a study conducted at Mumbai, India,^30^ which showed the reverse. The lower specificity of VILI when compared with VIA in this study may have been caused by cervicitis and immature metaplasia, particularly in postmenopausal women, which may be taken as VILI-positive. The higher sensitivity achieved when the Papanicolaou test cytology is combined with VILI when compared with Papanicolaou test cytology combination with VIA and each of the individual tests is similar to the reports of similar studies.^2,27,30,31^ The PPV of any diagnostic tool varies according to the peculiarities of the population; thus, it should not be used as a determinant of the accuracy of a diagnostic tool.^26^ The results of this study support the findings of Sankaranarayanan et al,^27^ which showed that the combination of VIA with Papanicolaou test cytology improved the overall accuracy of the screening procedures. The increasing trend of cervical neoplasia with age in this study is in support of the natural history of cervical cancer: it can take approximately 7 to 20 years for cervical intraepithelial neoplasia to transform to cervical cancer,^32^ and it can take several years for the human papillomavirus infection to transform to cervical tumor.

Strengths of this study were that the procedures—Papanicolaou test cytology, VIA, VILI, and histology—were carried out by qualified personnel and that the VIA and VILI groups were selected through randomized sampling. A weakness of the study was that the punch biopsies were not directed by colposcopy. This might have caused us to miss some participants who were truly positive. The authors’ inability to perform human papillomavirus testing in this study may have affected the histology report of some of the samples. Because this was a resource-limited setting, this study was also weakened by inadequate facilities for quality assurance. This was also a hospital-based study, in which the findings may not be a true reflection of what is obtainable in society.

In conclusion, this study showed that the overall accuracy of Papanicolaou test cytology, VIA, and VILI were essentially similar. VIA can also complement Papanicolaou test cytology in cervical cancer screening. Therefore, VIA or VILI can be used as a stand-alone cervical cancer screening test when compared with Papanicolaou test cytology, especially in low-resource settings. Effort should be geared toward training health care providers in the use of VIA and VILI in this environment. Larger community-based multicenter studies are required on this subject matter to validate or refute the findings of this study.
